# RETRACTION: Comparison of Wound Complications Between One‐Stage and Two‐Stage Brachiobasilic Arteriovenous Fistula: A Meta‐Analysis

**DOI:** 10.1111/iwj.70342

**Published:** 2025-03-17

## Abstract

**RETRACTION:** LuY. , XiaoJ. , LiuC. , and WangY. , “Comparison of Wound Complications Between One‐Stage and Two‐Stage Brachiobasilic Arteriovenous Fistula: A Meta‐Analysis,” International Wound Journal 20, no. 9 (2023): 3786–3793, 10.1111/iwj.14278.37337468 PMC10588325

The above article, published online on 19 June 2023, in Wiley Online Library (http://onlinelibrary.wiley.com/), has been retracted by agreement between the journal Editor in Chief, Professor Keith Harding; and John Wiley & Sons Ltd. Following an investigation by the publisher, all parties have concluded that this article was accepted solely on the basis of a compromised peer review process. The editors have therefore decided to retract the article. The authors did not respond to our notice regarding the retraction.

**RETRACTION:** Y. Lu , J. Xiao , C. Liu , and Y. Wang , “Comparison of Wound Complications Between One‐Stage and Two‐Stage Brachiobasilic Arteriovenous Fistula: A Meta‐Analysis,” International Wound Journal 20, no. 9 (2023): 3786–3793, 10.1111/iwj.14278.37337468 PMC10588325

The above article, published online on 19 June 2023, in Wiley Online Library (http://onlinelibrary.wiley.com/), has been retracted by agreement between the journal Editor in Chief, Professor Keith Harding; and John Wiley & Sons Ltd. Following an investigation by the publisher, all parties have concluded that this article was accepted solely on the basis of a compromised peer review process. The editors have therefore decided to retract the article. The authors did not respond to our notice regarding the retraction.

